# PlantPAD: a platform for large-scale image phenomics analysis of disease in plant science

**DOI:** 10.1093/nar/gkad917

**Published:** 2023-10-28

**Authors:** Xinyu Dong, Kejun Zhao, Qi Wang, Xingcai Wu, Yuanqin Huang, Xue Wu, Tianhan Zhang, Yawen Dong, Yangyang Gao, Panfeng Chen, Yingwei Liu, Dongyu Chen, Shuang Wang, Xiaoyan Yang, Jing Yang, Yong Wang, Zhenran Gao, Xian Wu, Qingrong Bai, Shaobo Li, Gefei Hao

**Affiliations:** State Key Laboratory of Public Big Data, College of Computer Science and Technology, Guizhou University, Guiyang 550025, China; State Key Laboratory of Public Big Data, College of Computer Science and Technology, Guizhou University, Guiyang 550025, China; State Key Laboratory of Public Big Data, College of Computer Science and Technology, Guizhou University, Guiyang 550025, China; Department of Computer Science and Technology, Tsinghua University, Beijing 100084, China; Text Computing & Cognitive Intelligence Engineering Research Center of National Education Ministry, Guizhou University, Guiyang 550025, Guizhou, China; State Key Laboratory of Public Big Data, College of Computer Science and Technology, Guizhou University, Guiyang 550025, China; National Key Laboratory of Green Pesticide, Key Laboratory of Green Pesticide and Agricultural Bioengineering, Ministry of Education, Center for Research and Development of Fine Chemicals, Guizhou University, Guiyang 550025, China; Center for Research and Development of Fine Chemicals, Guizhou University, Guiyang 550025, China; National Key Laboratory of Green Pesticide, Key Laboratory of Green Pesticide and Agricultural Bioengineering, Ministry of Education, Center for Research and Development of Fine Chemicals, Guizhou University, Guiyang 550025, China; Center for Research and Development of Fine Chemicals, Guizhou University, Guiyang 550025, China; State Key Laboratory of Public Big Data, College of Computer Science and Technology, Guizhou University, Guiyang 550025, China; National Key Laboratory of Green Pesticide, Key Laboratory of Green Pesticide and Agricultural Bioengineering, Ministry of Education, Center for Research and Development of Fine Chemicals, Guizhou University, Guiyang 550025, China; Center for Research and Development of Fine Chemicals, Guizhou University, Guiyang 550025, China; National Key Laboratory of Green Pesticide, Key Laboratory of Green Pesticide and Agricultural Bioengineering, Ministry of Education, Center for Research and Development of Fine Chemicals, Guizhou University, Guiyang 550025, China; Center for Research and Development of Fine Chemicals, Guizhou University, Guiyang 550025, China; State Key Laboratory of Public Big Data, College of Computer Science and Technology, Guizhou University, Guiyang 550025, China; National Key Laboratory of Green Pesticide, Key Laboratory of Green Pesticide and Agricultural Bioengineering, Ministry of Education, Center for Research and Development of Fine Chemicals, Guizhou University, Guiyang 550025, China; Center for Research and Development of Fine Chemicals, Guizhou University, Guiyang 550025, China; National Key Laboratory of Green Pesticide, Key Laboratory of Green Pesticide and Agricultural Bioengineering, Ministry of Education, Center for Research and Development of Fine Chemicals, Guizhou University, Guiyang 550025, China; Center for Research and Development of Fine Chemicals, Guizhou University, Guiyang 550025, China; National Key Laboratory of Green Pesticide, Key Laboratory of Green Pesticide and Agricultural Bioengineering, Ministry of Education, Center for Research and Development of Fine Chemicals, Guizhou University, Guiyang 550025, China; Center for Research and Development of Fine Chemicals, Guizhou University, Guiyang 550025, China; National Key Laboratory of Green Pesticide, Key Laboratory of Green Pesticide and Agricultural Bioengineering, Ministry of Education, Center for Research and Development of Fine Chemicals, Guizhou University, Guiyang 550025, China; Center for Research and Development of Fine Chemicals, Guizhou University, Guiyang 550025, China; State Key Laboratory of Public Big Data, College of Computer Science and Technology, Guizhou University, Guiyang 550025, China; Department of Plant Pathology, Agriculture College, Guizhou University, Guiyang 550025, Guizhou, China; New Rural Development Research Institute, Guizhou University, Guiyang 550025, Guizhou, China; National Key Laboratory of Green Pesticide, Key Laboratory of Green Pesticide and Agricultural Bioengineering, Ministry of Education, Center for Research and Development of Fine Chemicals, Guizhou University, Guiyang 550025, China; Center for Research and Development of Fine Chemicals, Guizhou University, Guiyang 550025, China; National Key Laboratory of Green Pesticide, Key Laboratory of Green Pesticide and Agricultural Bioengineering, Ministry of Education, Center for Research and Development of Fine Chemicals, Guizhou University, Guiyang 550025, China; Center for Research and Development of Fine Chemicals, Guizhou University, Guiyang 550025, China; State Key Laboratory of Public Big Data, College of Computer Science and Technology, Guizhou University, Guiyang 550025, China; State Key Laboratory of Public Big Data, College of Computer Science and Technology, Guizhou University, Guiyang 550025, China; National Key Laboratory of Green Pesticide, Key Laboratory of Green Pesticide and Agricultural Bioengineering, Ministry of Education, Center for Research and Development of Fine Chemicals, Guizhou University, Guiyang 550025, China; Center for Research and Development of Fine Chemicals, Guizhou University, Guiyang 550025, China

## Abstract

Plant disease, a huge burden, can cause yield loss of up to 100% and thus reduce food security. Actually, smart diagnosing diseases with plant phenomics is crucial for recovering the most yield loss, which usually requires sufficient image information. Hence, phenomics is being pursued as an independent discipline to enable the development of high-throughput phenotyping for plant disease. However, we often face challenges in sharing large-scale image data due to incompatibilities in formats and descriptions provided by different communities, limiting multidisciplinary research exploration. To this end, we build a Plant Phenomics Analysis of Disease (PlantPAD) platform with large-scale information on disease. Our platform contains 421 314 images, 63 crops and 310 diseases. Compared to other databases, PlantPAD has extensive, well-annotated image data and in-depth disease information, and offers pre-trained deep-learning models for accurate plant disease diagnosis. PlantPAD supports various valuable applications across multiple disciplines, including intelligent disease diagnosis, disease education and efficient disease detection and control. Through three applications of PlantPAD, we show the easy-to-use and convenient functions. PlantPAD is mainly oriented towards biologists, computer scientists, plant pathologists, farm managers and pesticide scientists, which may easily explore multidisciplinary research to fight against plant diseases. PlantPAD is freely available at http://plantpad.samlab.cn.

## Graphical Abstract

**Graphical Abstract:**
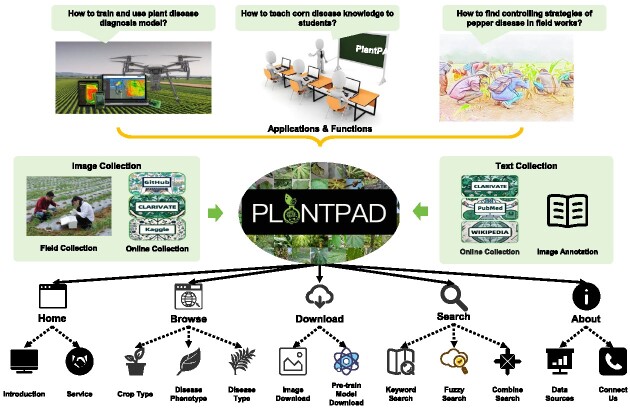

## Introduction

In the coming decades, the need to feed a burgeoning population is among the most pressing challenges that humanity will confront, and this challenge is aggravated by plant diseases. According to the United Nations, the population of the world will reach 9.6 billion by 2050 (1–3). Plant disease can lead to losses ranging from 10% to 100% in certain cases, afflicting individual households to entire regions (4). Through the implementation of phenomics-based intelligent agronomic technologies, it is possible to mitigate 100% of yield losses associated with undetected plant disease (5). This intervention could potentially enable the provision of sustenance to an additional 0.41 billion people, accounting for approximately 5.26% of the global population in 2020 (6,7). Therefore, managing plant disease is crucial for safeguarding crops and ensuring a stable food supply.

Phenotype-based diagnosis, a rapid and convenient method, is crucial for plant disease management. When a plant is infected by a pathogen, it usually displays visible signs or even damage to its tissues, and thus this phenotype is the most common and useful criteria for the diagnosis of plant disease (8–10). Expert recognition and machine learning are two major measures for phenotype analysis. The former is a traditional method that requires trained specialists to visually examine the shape, color and texture of diseased leaves or other tissue and match them with known disease images or descriptions (11–13). The latter is a contemporary method utilizing computer approaches such as deep learning that can learn from image data and automatically classify diseased leaves or other issues by analyzing their features (14–16). The development of the image phenomics of plant diseases can not only facilitate the training of specialists, but also enhance the performance of machine learning algorithms through the provision of relevant and informative features of disease patterns (17–19). Hence, phenomics is being pursued as an independent discipline to enable the development of high-throughput phenotyping for plant diseases.

Recently, phenomics technologies have made great progress in managing plant diseases. French et al. adopted effective plant phenomics analysis to explore the communication between diseased biomass and the environment, improving precision microbiome management of plant disease (20). Antoin et al. noted that artificial intelligence (AI) offered unprecedented opportunities for computational data analysis in digital plant phenology and would have an extraordinary impact on plant science in the coming decades (21). Phenomics technology is generating enormous quantities of next-generation plant image data (22–24). For this reason, scientists have gradually begun to create image databases of plant phenotyping, such as PlantVillage (25), Planteome (26) and similar works. We provide a brief overview of some notable datasets and compare their amounts of data, as shown in Table 1. These plant phenotyping image datasets also provide resources for efficient phenomics analysis of plant disease. For instance, Fan *et al.* utilized two apple leaf databases as well as a coffee leaf database to train plant disease diagnostic models and obtain discriminative plant phenotypic features (27). Zhao *et al.* designed and implemented a plant disease diagnostic model with generalization using three databases for tomatoes and potatoes (28). However, researchers often face challenges and limitations in sharing these data, due to incompatibilities in data formats, descriptions of observations, size of data and annotation completeness, inhibiting multidisciplinary research exploration.

**Table 1.** Some representative databases of plant diseases

Database name	Species number	Diseases number	Classes number	Image number
IDADP Database^a^	Unspecific	Unspecific	Unspecific	Over 200 000
The Challenger 2018 Pest and Disease Classification Dataset^b^	10	27	63	Over 50 000
Plant-Village Dataset (25)	14	26	38	54 303
Rice Leaf Disease Image Sample Dataset (29)	1	4	4	5932
LWDCD Dataset (30)	1	9	10	Over 12 000
Leaflet Cassava Dataset (31)	1	1	3	2756
The Kaggle Challenger Cassava Disease Dataset^c^	1	4	5	22 031
Plant Pathology 2021-FGVC8 Dataset^d^	1	12	12	23 000
Crop Pest Dataset (32)	4	40	40	600
IP102 Dataset (33)	Unspecific	102	102	Over 75 000
PlantDoc Dataset (34)	13	Unspecific	17	2598
PDD Dataset (35)	42	271	271	220 592

^a^ http://www.icgroupcas.cn/website_bchtk/index.html.

^b^ https://aistudio.baidu.com/aistudio/datasetdetail/76075.

^c^ https://www.kaggle.com/c/cassava-disease.

^d^ https://www.kaggle.com/c/plant-pathology-2020-fgvc7.

To address this issue, we built a dataset, called the Plant Phenomics Analysis of Disease (PlantPAD), including 421 314 images, 63 crops and 310 diseases. PlantPAD is a comprehensive tool that uses image data and text information to identify and analyze plant diseases, providing valuable support for agricultural production, education and training, and scientific research. Our image database for plant disease integrates data and models to provide convenient, fast, accurate and professional plant disease identification and analysis. Its efficient and useful information on plant diseases can serve various purposes. Our database has several advantages.

- First, it has richer and more comprehensive image data for plant diseases, meeting the multidisciplinary needs of various users.
- Second, it provides detailed image annotation and various disease-related information, which helps users obtain more knowledge concerning plant disease and improve their management skills.
- Third, our database also incorporates pre-trained deep-learning models for plant disease diagnosis, improving working efficiency.

## Database

### Data type and source

**Figure 1.**
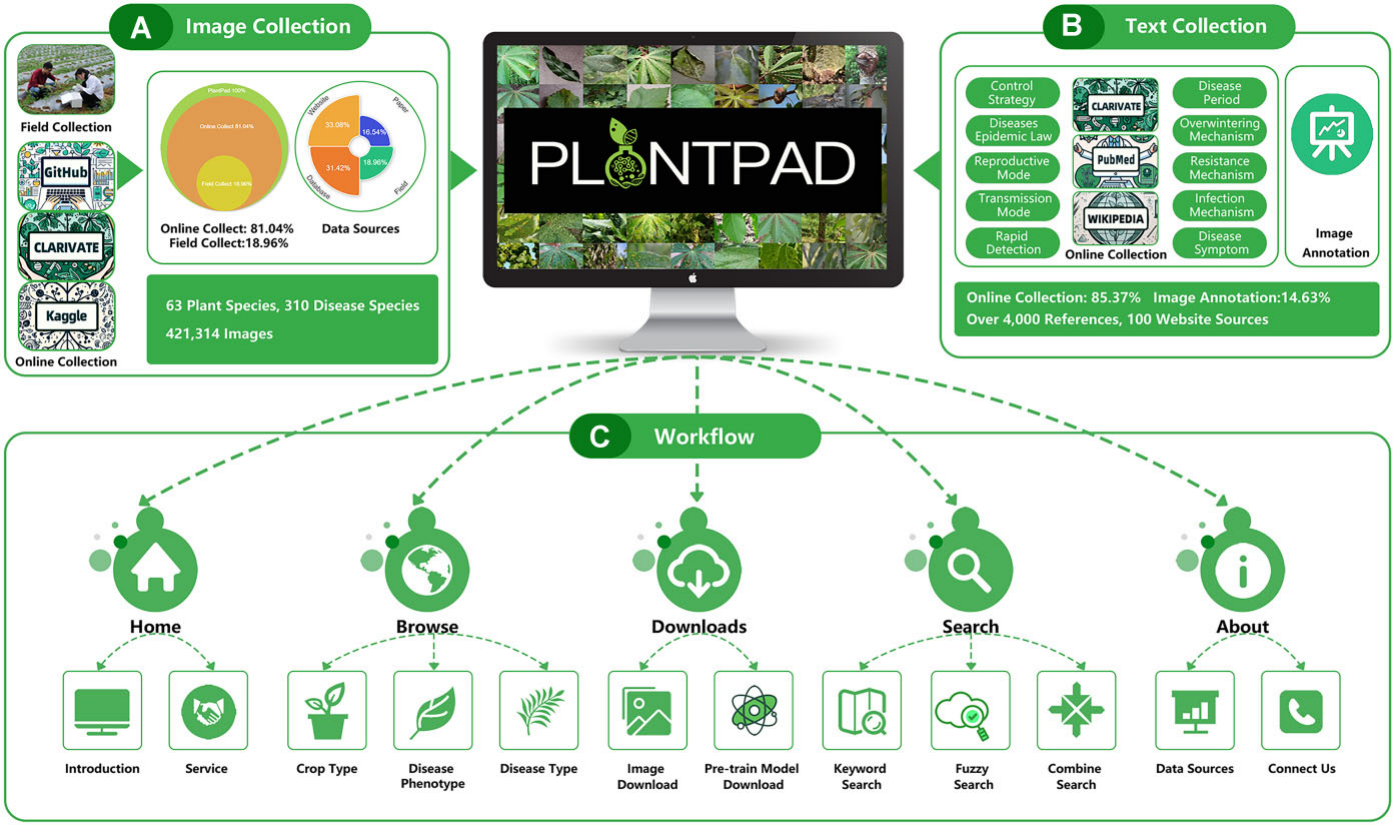
Overview of image collection, text collection and workflow for PlantPAD. (**A**) Main image collection methods for PlantPAD and distribution of image data. PlantPAD data is collected mainly through field collection and web collection, where field collection accounts for 18.96% of the data and web collection accounts for 80.04%. Furthermore, the distribution of the sources of our data can be grouped into four parts: 18.96% field collection, 33.08% other websites, 31.42% other databases and 16.54% paper collection. (**B**) The primary methods for collecting text data for PlantPAD include network collections and image annotation, with network collection accounting for 85.37% of the data and image text annotation accounting for the remaining 14.63%. (**C**) PlantPAD’s main workflow includes five main functions: home, browse, downloads, search and about, each with some sub-functions.

PlantPAD offers two main data types: image and text, as shown in Figure 1A and B. The image data, including 421 314 images for 63 plant species and 310 disease phenotypes, is derived from two sources: online labeled datasets and field data captured by our team. The text data contains manual annotations of images and disease-related information from online collection, derived from over 4000 references and 100 websites.

#### Image information collection

In the collected data, 81.04% of images are from online sources, 33.08% from websites, 31.42% from other databases and 16.54% from reference works. In addition, 18.96% of images are drawn directly from the field, as shown in Figure 1A. For the online collection, we begin by conducting an extensive search for open-source datasets that are available on reputable platforms and research repositories. These sources include agricultural research institutes, academic institutions and government agencies focusing on plant diseases and related fields. We also take into account factors such as the source and size of the database, its reputation and credibility of the organization or researchers involved, the number of disease categories included and the accuracy and consistency of the labeling and annotation. Second, for field collection, to ensure that each image in our dataset was labeled accurately and consistently, we invited seven agricultural experts to join the dataset construction and to tag images according to classification and naming guidelines. During the tagging, metadata such as the time, location, species and environment of each image was recorded to ensure that the dataset had sufficient quantity, diversity and accuracy to facilitate subsequent data processing and analysis. Additionally, due to the uncertainty and fragmentation of the time and location of disease occurrence, we adopt a combination of field photography and online collection to increase the amount and diversity of our data. Finally, we obtain the full image dataset combined with online and field collection.

To account for the disease patterns of different plants, we set up a hierarchical classification system based on plant species and common disease types to label and organize the dataset. This system consists of a root catalog, plant type catalog, plant species catalog and disease sub-catalog. For instance, for rice leaf blight, the corresponding directory structure is /staple crops/rice/leaf blight/. To keep the data in our database current and reliable, we follow a regular update schedule, constantly adding new images and relevant information to reflect the latest findings and the current plant disease landscape.

#### Text information collection and image annotation

Our text data consists of two types: 85.37% of the disease-related information is collected from the Internet and 14.63% of the descriptive information is from our annotations to accompany the images, as shown in Figure 1B. Both types of textual data have undergone rigorous quality control and management to ensure accuracy, completeness and consistency. The collection details are as follows.

In order to meticulously gather disease-related information, we employed a methodical and systematic approach. Firstly, we diligently sourced data from credible and reliable sources, such as specialized literature, research reports, agricultural platforms and other reputable online resources. Secondly, we meticulously evaluated and assessed the data we collected to ensure its utmost accuracy and consistency. Our screening criteria encompassed both basic and advanced information, which was of utmost importance for accurately identifying and effectively controlling plant diseases based on factors such as crop type, disease mode, spread and prevalence, occurrence cycle, environmental conditions, overwintering mode, control strategies, disease symptoms, and signs. We sourced advanced information from specialized literature on plant diseases, encompassing topics such as resistance, rapid detection methods, infection mechanisms, and potential targets. More authoritative and comprehensive sources of information were considered in the selection process for disease data, with professional literature receiving the highest priority. This is due to the recognized comprehensiveness and systematic nature of knowledge found in review papers within professional literature, while research papers provide cutting-edge and specific findings. Official reports from professional bodies and reports from research institutes were also deemed relevant sources and were given second priority. Subsequently, agricultural information platforms and reputable online resource platforms were considered the lowest priorities in terms of data selection. When there are multiple sources of diverse information on plant diseases or conflicting information on plant diseases, our website selects data from the highest-priority sources to obtain valuable and relevant information. Generally, we employ a variety of methods to ensure that the data collected aligns with the objectives and scope of the database, ultimately enabling us to provide users with highly relevant disease data.

Within PlantPAD, three distinct categories of descriptive information pertaining to plant disease images exist. Firstly, we have the image metadata, encompassing fundamental attributes shared by all images. This comprises data on the image’s source, format, dimensions, resolution and sharpness. These metadata are vital for providing a foundational understanding of each image. Secondly, textual annotations represent another critical facet. These annotations are meticulously structured textual data, tailored to align with the common features and search criteria used in plant disease identification. They serve as a valuable resource for users, facilitating swift access to plant diseases of interest or those requiring diagnosis. Lastly, an innovative data type is introduced, involving textual descriptions of features exhibited within the images. These descriptions encompass various aspects such as disease morphology, coloration and spatial location. They function to augment and enrich the image data, offering insights that may be challenging to convey through visual elements alone. This augmentation enhances data diversity and completeness. To ensure the uniformity and precision of textual detail descriptions, we have implemented a methodical approach, establishing a meticulous annotation manual. This comprehensive manual outlines specific criteria for annotating plant disease images, encompassing parameters like disease color, morphology, extent of damage, and precise capture location. To accommodate the diverse array of crop types and disease categories within our repository, we have designed specialized image annotation software. This software adheres to the guidelines laid out in the annotation manual, expediting the annotation process while simultaneously enhancing the quality of results. Through these measures, we have successfully amassed a substantial volume of disease-related textual data, offering comprehensive coverage of various facets related to plant disease images. This approach ensures consistency, accuracy, and usability in our extensive dataset, fostering more effective research and analysis in the realm of plant pathology.

In total, the final text information is sorted in combination with disease-related and image annotation information. In detail, for every plant disease, there are ten categories of text information, as shown in Figure 1B. For each image, there are two categories of annotation information, including the context of the disease phenotype and meta-information for the image. Through a stringent screening process, we filter relevant text information and transform it to fit our database’s format and standards. In addition, we ensure optimal balance and adequacy within our textual data for each category, taking into account their significance and accessibility.

### Workflow

The workflow of PlantPAD incorporates several key components, as shown in Figure 1C. First, users can access the database’s homepage to gain insight into its fundamental aspects, including an introduction to the database and some easily used services. Next, the browse function empowers users to explore plant disease images using a range of classification methods, such as crop type, disease type, or disease phenotype. This streamlined approach facilitates the swift discovery of relevant images, catering to the user’s specific interests. In addition to providing a convenient download function and disease-specific image downloads, PlantPAD also provides PDDD-PreTrain, a plant disease pre-training model based on deep learning technology. PDDD-PreTrain is a series of pre-trained models for plant disease diagnosis, which capitalizes on the richness and diversity of a large-scale plant disease dataset, and by learning more specialized and applicable plant disease features and knowledge from the data set, it improves the generalization ability and robustness of the models. Experimental results show that PDDD-PreTrain can improve the accuracy and efficiency of diagnosis in multiple plant disease diagnosis subtasks. Furthermore, users can employ the search function to pinpoint images of plant disease by inputting keywords, fuzzy terms and combined search approaches. Finally, for the related function, users can access valuable insights into the database’s data sources and contact details for the development team.

### Data statistics

**Figure 2.**
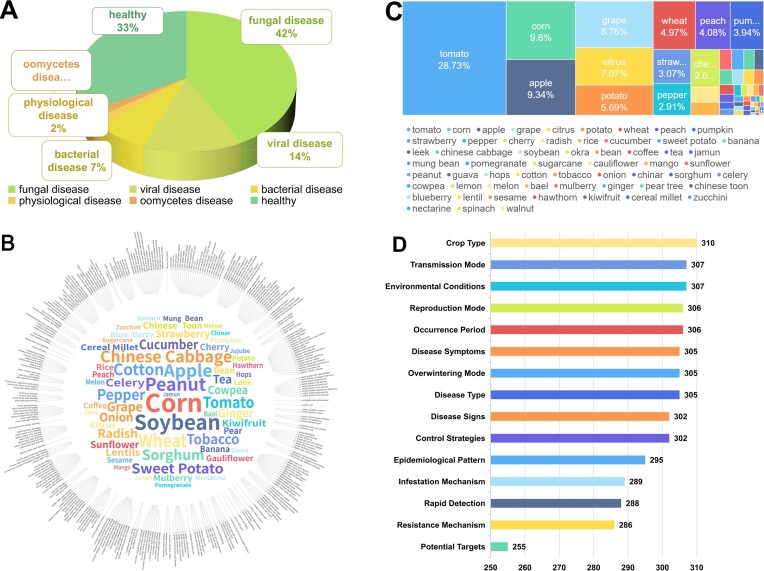
Statistics of the PlantPAD. (**A**) Distribution of data across the database, including the healthy category and five common pathogen categories. Statistics are compiled on the percentage of images in the dataset, by pathogen category, with 42% fungal images, 33% healthy images, 14% virus images, 7% bacterial images, 2% physiological disease images and 2% oomycete images. (**B**) Distribution of data across the database according to the category of plant diseases. The number of images of the 63 plant species in the dataset is statistically organized, and it can be found that the number of images creates a natural long-tailed distribution according to how common they are in nature. (**C**) All plant species and categories of plant diseases are in the database. By categorizing by crop type, plant type and plant disease type to obtain a dendrogram containing 63 plant species, and 310 diseases, with a word cloud in the middle based on disease types. For detailed information on each disease category in the database, please refer to the supporting material. (**D**) Summary of textual data on all plant diseases in the database. Crop type, disease type, mode of reproduction, epidemiological pattern and mode of transmission, period of occurrence, environmental conditions, overwintering mode, control strategies, disease symptoms, disease signs, resistance mechanisms, rapid detection methods, infestation mechanisms and potential targets of 310 diseases are collected in academic journals and authoritative websites, with the total number of disease categories counted.

Here, we provide statistics for specific proportions of image data for healthy and disease subjects, as shown in Figure 2A. In detail, 33% of images are of healthy plants and 67% are of diseases, including 42% fungal disease, 14% viral disease, 7% bacterial disease, 2% oomycetes disease and 2% physiological disease. Fungal diseases, which are notorious for the considerable harm they inflict on plants, stand out prominently. Due to the wide range of pathogenic types caused by fungal diseases (36), fungal disease visuals are 42% of all images, markedly surpassing other categories.

To ensure the richness and applicability of the database, we have categorized plant disease images, covering a diverse array of plant species, including 63 plants and 310 distinct disease types, as depicted in Figure 2B. In addition, we provide a plant word cloud diagram in accordance with the number of disease types in the center of Figure 2B. From the tree diagram, different crop branches account for various numbers of disease types. Due to space limitations on the paper, the specific number of images related to each disease can be found in Tables S1–S4, which are included in the supplementary data. Field grain crops and vegetables can be affected by more pathogens than fruit trees. The cloud diagram shows that corn has the most types of plant diseases relative to other crops in our database. This phenomenon may be a result of the host range of pathogens, completeness of data collection and plant area.

To more clearly show the distribution of disease images, we analyze and visualize the 63 plant disease image numbers in our database, as shown in Figure 2C. We find that a distinct, natural, long-tailed distribution pattern emerges. This pattern is characterized by the prevalence of longer tails and shorter heads relative to image numbers. In simpler terms, a few categories contain a significantly higher number of images than the remaining categories. For instance, it is evident that images of tomato diseases are numerically dominant, encompassing 28.7% of all disease images in the database. The abundance of images for tomato diseases may result from the research hotspots, plant area and time of the first study.

We also report the results of text information statistics, as shown in Figure 2D, which indicates the distribution of text data for each disease category in the database. Information items on environmental conditions have the second highest number of all types of information, which also indicates that many studies focused on environmental conditions. The least amount of text is for potential target information, and we speculate that there are many disease targets that have yet to be found.

### Web design

#### Frontend

Using the Vue framework, we develop a frontend interface for the plant and disease datasets, capitalizing on its component-based approach and responsive design. The interface enables users to browse crop types and disease categories to retrieve pertinent information on plants and diseases through the classification and search features. The data includes three main crop types: staple crops, economic crops and horticultural crops, which users can access using labeled buttons. Users can also examine specific disease types, including fungal diseases, bacterial diseases, viral diseases, oomycete diseases and physiological diseases by selecting relevant labels. In addition, the interface allows users to conduct searches based on attributes such as color, texture and leaf morphology, augmenting the precision of information retrieval. Overall, the Vue-powered frontend provides an immersive and user-friendly experience, enabling users to seamlessly explore and comprehend diverse traits of diseases, the plants they afflict and their impacts.

#### Backend

Using MySQL 8.0.12 and Tencent Cloud OS, we develop a backend system for PlantPAD that responds to requests from the frontend interface and retrieves pertinent data from the database. This database contains vital information on plants and diseases, such as crop types, disease types and various attributes. The backend processes users’ selections and search criteria, filtering and extracting the data in response. The backend also supports the uploading of images and files to Tencent Cloud OS and supplies file links for the frontend interface. The backend system ensures data security, stability and efficiency, delivering accurate and rapid data support to the frontend interface. The system also capitalizes on Tencent Cloud OS’s stability and scalability, effectively managing and storing a vast amount of data and files, ensuring the reliability and accessibility of data.

### Website functions and usage

**Figure 3.**
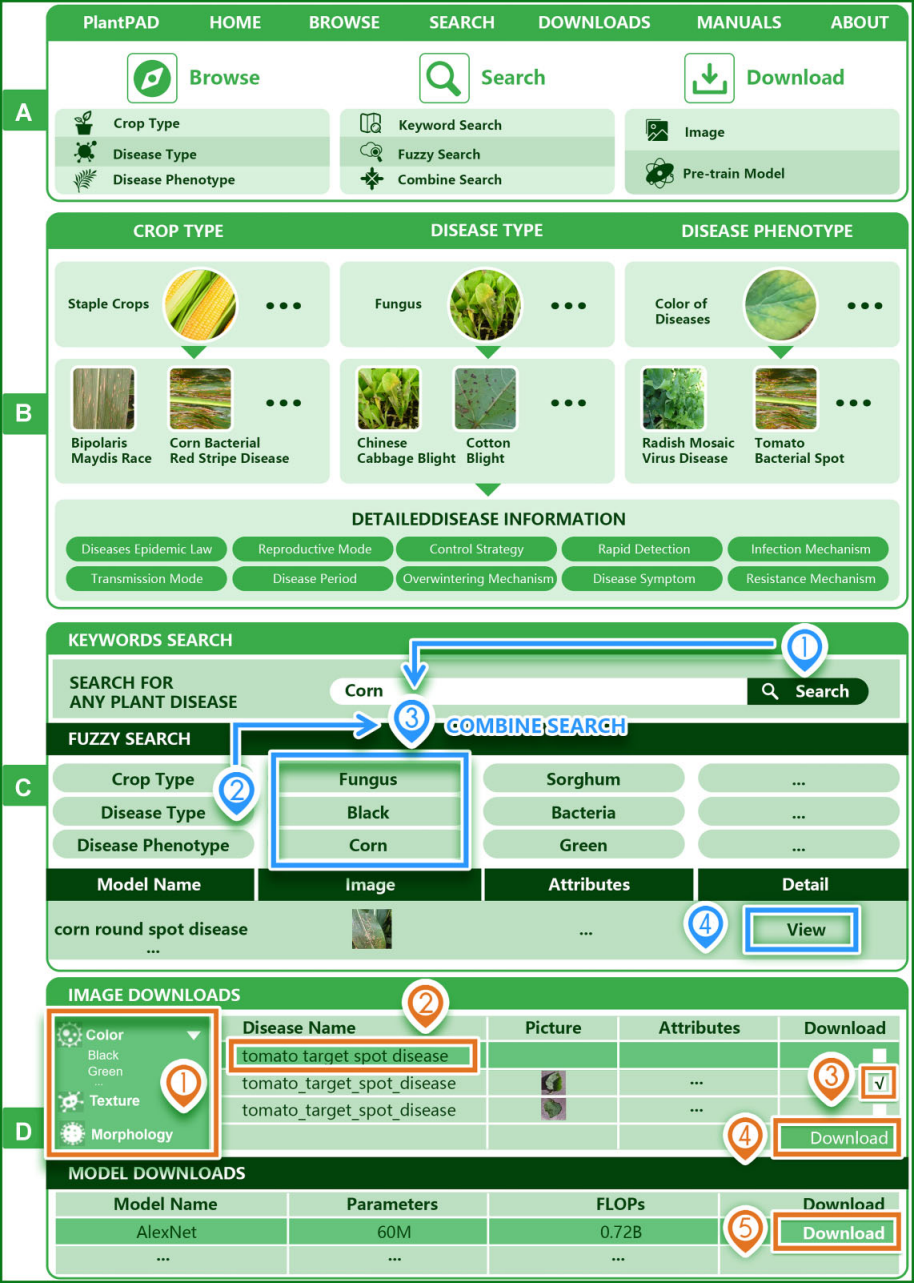
Website design for the PlantPAD. (**A**) The introduction to the main functions of Browse, Search and Download in the database. (**B**) An example of the operation of the browse function in the database. Browsing can be done by crop type, disease type and disease phenotype to get the required detailed information about the disease. (**C**) An example of the operation of the search function in the database. Three different search methods, namely, keyword search, fuzzy search and combination search, can be used to obtain the required disease details and disease pictures. (**D**) An example operation of the downloads function in the database. Specific ways to download images and pretrain models are illustrated.

PlantPAD is a free online database that allows users to browse, search and download plant disease data and AI models. Users can browse data by crop type, disease type, or phenotype, search data by keywords or combinations, and download images and pre-trained models, as shown in Figure 3A. The manual page and the About page provide instructions and information on PlantPAD’s team, sources and contacts. Overall, the comprehensive functions and simple use of PlantPAD make it easier for users to conduct research using plant disease data.

#### Browse

PlantPAD allows users to browse data on crop types, disease types and disease phenotypes. Users can select any of these options from the browse page in the navigation bar. For example, selecting crop types leads to a page with three blocks of different crops, each with an image album. Users can click on any crop image to see a list of diseases related to that crop, and click on any disease image to see detailed information on that disease. The same process applies to the other two options of disease types and disease phenotypes as shown in Figure 3B. In sum, PlantPAD provides complete data browsing function, making it convenient for users to use plant disease data.

#### Search

The search page of PlantPAD enables users to retrieve plant disease information quickly by using three functions: keyword search, fuzzy search and combined search, as shown in Figure 3C. The keyword search matches entries with complete or partial keywords in a drop-down menu. The fuzzy search filters entries by crop type, disease type, or phenotype. The combined search uses both keyword and fuzzy search to refine the results. Users can click on the links in the drop-down menu to access more details on each entry. PlantPAD offers various search options for different levels of interest.

#### Downloads

PlantPAD allows users to download plant disease images and diagnosis models, as shown in Figure 3D. Users can filter images by color, texture, or morphology and select diseases from a drop-down menu. Users can also batch select and download images with a menu button. Users can download over 20 deep learning models for plant disease diagnosis. The download function helps users get the data they want easily.

#### Others

PlantPAD also provides functions to correct errors, upload data and contact the administrators, all integrated into the about page. Users can find our contact information here and enter feedback messages in the space provided, allowing us to receive error corrections or additional data to enrich PlantPAD.

## Database application

PlantPAD plays an important role in disease diagnosis, plant pathology teaching and disease man-agreement. To detail the introduction of the convenience and functionality of PlantPAD in the three aspects, we selected typical cases on fruit trees, grain and vegetables. The detailed operation process is displayed as follows.

### Case study 1: How to train and use pear disease diagnosis model in smart agriculture

The pear is an important fruit worldwide, providing a variety of nutrients and able to be used to make medical drugs (37). Pear growth is susceptible to more than 30 diseases, including ring rot, leaf rust, brown rot, anthracnose and brown spot, resulting in 30–70% losses of yield (38). At present, molecular diagnosis is the most commonly used detection technology for pear diseases, although it is complicated, expensive and unable to achieve timely diagnosis (39,40). It is crucial, therefore, to develop the smart pear disease diagnostic technology to improve the efficiency of the disease control.

**Figure 4.**
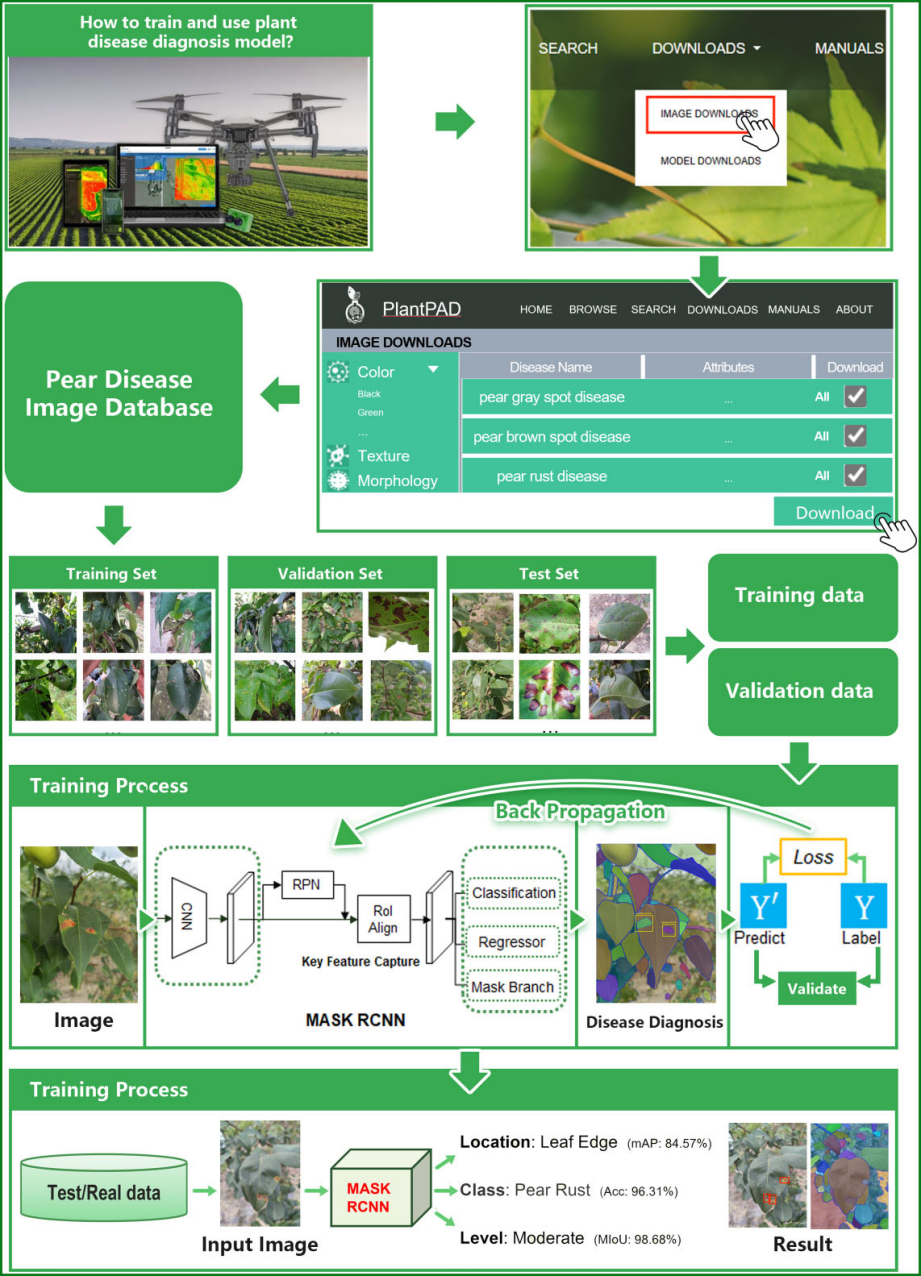
The case for training and testing plant disease diagnostic models using PlantPAD. First, users can choose the pear disease image data from PlantPAD to build a comprehensive pear disease database as an available dataset. Then, the dataset can be divided into a training set, validation set and test set, and the data can be preprocessed to derive the essential training and validation data needed for model training. Subsequently, the Mask RCNN model was trained to obtain the requisite disease information, such as category, location and disease level necessary for effective diagnosis of plant diseases. In detail, users can adopt the loss function and backpropagation algorithms to enhance the accuracy of the model until the data for the validation of performance no longer improves. Last, once the model training is complete, the well-trained model can be leveraged to diagnose plant diseases on real data or test data.

In particular, users can utilize PlantPAD to use classification, detection and a segmentation model to diagnose pear disease, as shown in Figure 4. First, they can acquire disease image data on the image download interface. Then, the pear images are separated into training sets to train a disease diagnosis model, validation sets to verify the effectiveness of the predicted results, and test sets to test the diagnostic performance for the unseen plant disease image. In particular, the users download image data on pear diseases from PlantPAD and compile a pear disease database to obtain training and validation data for the plant disease diagnostic model. The training and validation sets undergo preprocessing, including data cleaning or augmentation, before being fed into the model for training. During the training process, the images are transformed into discriminative plant disease phenotypic feature maps using convolutional neural networks (41,42), which are then further processed to capture key features by the RPN and ROI align module. In addition, these features are sent to the classifier for disease category determination, the regressor to obtain disease location, and the mask branch for the quantification of disease levels. Back propagation is used to continuously optimize the model’s performance through comparison of its results with those of the validation data. Finally, the trained pear disease model can be used to identify pear diseases in real-world situations drawing on images from real data. As such, PlantPAD has the potential to aid in the development of disease diagnostic models for pear crops, facilitating the speedy identification, localization and classification of various pear diseases.

Currently, PlantPAD serves as a reliable source of data for the design and implementation of models for diagnosing plant diseases. However, certain issues remain to be resolved in PlantPAD. (i) The distribution of data in PlantPAD is non-uniform, making it challenging to obtain effective and discriminative plant phenotypic features for diagnosing plant diseases using insufficient image data. (ii) The poor quality of images, including out-of-focus and blurry ones and the paucity of image data for certain classes, such as jujube and walnut, may adversely impact the performance of the diagnostic model. (iii) While PlantPAD includes image annotations for plant disease categories, these lack further refinement, including the location and extent of the disease.

### Case study 2: how to teach corn disease knowledge to students

Corn, one of the most important cereals in the world, provides at least 30% of the energy needed for survival (43). As populations continue to increase dramatically, demand for corn in developing countries is expected to double by 2050 (44). Across all of corn’s growth stages, it can be affected by more than 80 diseases (45).The area across which corn disease is spread reaches about 600 million acres, causing 10–25% yield losses annually (45). Therefore, improved knowledge of corn diseases, including pathogens, regular epidemics and their interactions with plants will be beneficial to enabling and maintaining the sustainable development of corn planting.

**Figure 5.**
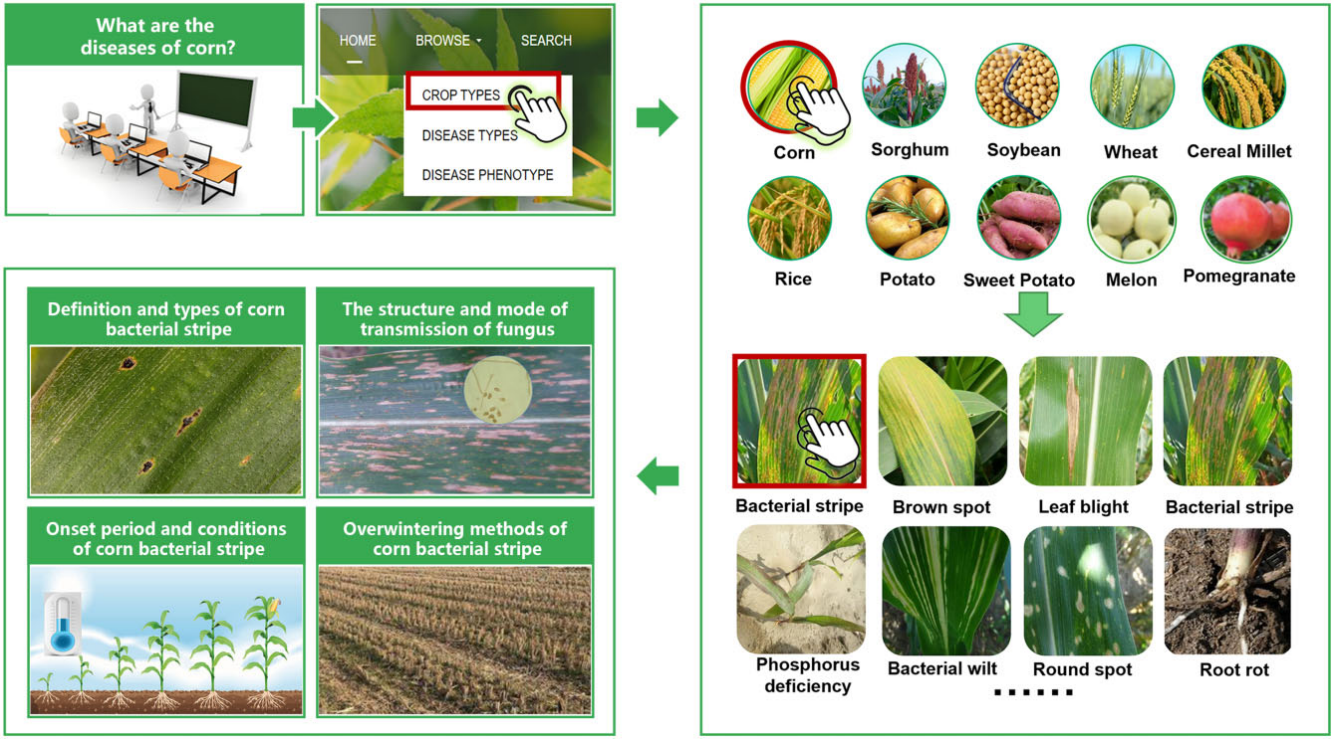
The case for the PlantPAD application in botany teaching. To illustrate instruction in corn diseases, first, the teacher can choose the crop category in the PlantPAD plant disease repository. Next, he/she can select the type of corn from the crop category and click on it. Then, the teacher can view various corn plant ailments once they are accessed, and look into the bacterial streak disease of corn to obtain a comprehensive analysis of the problem. Last, the student can identify the definition, type, structure, mode of contagion, onset period, conditions for survival, methods of overwintering in detail and quickly.

Generally, PlantPAD contains images and text information relative to corn diseases that can help teachers instruct students about these types of diseases, disease morphology and regular disease occurrence, using vivid materials, as shown in Figure 5. First, on the homepage of the database, the browse window is used to locate the crop types interface. There are three types of crops listed there: staple crops, horticultural crops and economic crops, of which corn belongs among the staple crops. After the crop category for corn is chosen, various diseases of corn can appear, such as bacterial stripe, brown spot, northern leaf blight, bacterial wilt and round post disease. Finally, by clicking on the bacterial strip, we can learn more about the definition and characteristics of the disease, structure and mode of transmission, onset periods and conditions, overwintering methods, methods of detection and mechanisms of infection, and other information. Corn bacterial stripe is caused by *Xanthomonas vasicola* pv. *vasculorum* which can lead to striped and brown disease lesions. For the onset period, diseases can occur at any stage of corn growth, but the symptoms are usually more visible after tasselling. This disease requires warm (25°C) and humid (90%) conditions for infection and symptom development. The disease can be spread by splashing rain, irrigation, wind, insects and contaminated equipment. Our database can provide the teaching purpose for corn diseases and help students have a deeper understanding of the life cycle, detection and prevalence of the diseases.

Overall, teachers can use PlantPAD to achieve the most basic teaching tasks, but there are also some challenges. (i) Deep knowledge of the mechanisms and signaling of pathogen infection, including R receptors and their corresponding effectors should be provided. (ii) The morphologies of pathogens and changes across different stages of infection should be collected. (3) The epidemic and regular incidence tendency of pathogens all around the world should be analyzed.

### Case study 3: how to find controlling strategies of pepper disease in field works

Pepper is an important cash crop worldwide, and its production is severely threatened by anthracnose disease (46).Pepper anthracnose can cause a yield loss of more than 40% (47). This disease is caused by the top 10 fungus *Colletotrichum* spp., which has complex lifestyle patterns, including necrotrophic, hemibiotrophic and latent stages, which improve its disease control (48,49). In practice, to obtain high-quality fruit, farmers tend to use fungicides >10 times. That not only to pollute the environment, but also increase costs (50). Therefore, it is urgently necessary to help field workers select suitable measures for effectively controlling pepper anthracnose.

**Figure 6.**
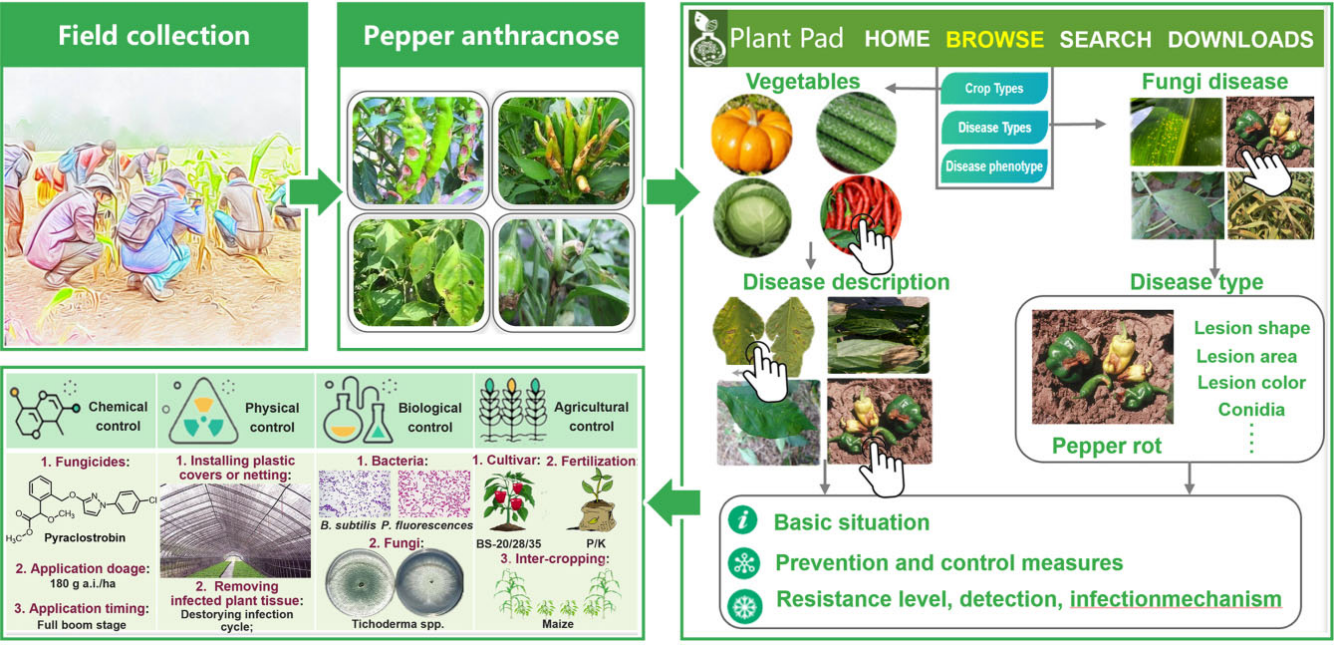
The case for the use of PlantPAD in the crop protection field. Using pepper anthracnose as an example, during field collection, necrotic lesions on pepper leaves and rhizomes can be observed with conidia of varying colors and concentric rings of perforations. To determine the type of disease, vegetables can be selected under the crop type section in the browse page of the database, and images of diseased chili peppers can be viewed for comparison. Furthermore, an infected pepper field can be identified by directly examining the disease type category. In addition, the database also provides comprehensive information on four measures for the control of pepper anthracnose, including chemical, physical, biological and agricultural methods.

PlantPAD can provide a systematic strategy and approach to anthracnose disease management across the whole growth of the pepper, as shown in Figure 6. First, field workers can capture images of pepper anthracnose occurring in fruits and leaves by locating sunken necrotic concentric rings, ring lesions, and conidia or acervuli. Then, to learn how to control this disease, from the browse page of the database, the user can click on crop types, select vegetables and view some pictures of diseases. After selecting pepper and comparing the images on the ‘disease description’ page, spots on pepper leaves and fruits are matched. Meanwhile, on the browse page, we can also find pepper anthracnose by looking up fungal disease. After finding disease types, pepper anthracnose can be determined according to the images and text descriptions. Finally, we obtain chemical, physical, biological and agricultural control measures that are available for use against pepper anthracnose. The chemical control measure pyraclostrobin at 180 g a.i./ha application at the full-bloom stage could significantly decrease anthracnose incidence (23.67%) and development (89.80%), delay anthracnose disease onset, and increase pepper yield by 10.7–29.2%. The measures for physical control include installing plastic covers or netting and removal of plant tissues, which can decrease disease propagation by wind or rain and impair the infection cycle. The application of biological bacteria (*B. subtilis*, *P. fluorescences*) and fungi (*Trichoderma* spp.) show satisfactory activity against anthracnose with an efficacy of 66.01–100%. For agricultural control, planting resistance cultivars including BS-20/28/35, the application of more phosphate and potassium fertilizers, and intercropping with corn at a ratio of 4 P/2 M can decrease the occurrence and development of pepper anthracnose. Our database can thus help field workers conveniently and quickly improve their knowledge and skills in pepper anthracnose control.

While PlantPAD is a useful resource for obtaining information on plant disease management, such as fungicide resistance, optimal dosage, application timing, biological responses to bacteria and fungi, resistance cultivars and intercropping, it has some limitations. (i) It only allows users to browse existing plant disease data and does allow them to upload their own images for automated identification; (ii) fungicide resistance distribution of disease pathogens all over the world has not been summarized and (iii) potential targets for new pesticide designs to realize efficient plant disease control should be collected. We plan to improve our database in the future to enable this function and enrich data sources.

## Deficiencies and prospects

The data for PlantPAD is already quite rich, but some aspects need to be improved. For example, (i) there are too few types of ornamental plants and medicinal plants, namely only three and if this part of the data can be supplemented, it will attract more users from different fields, such as horticulturists and pharmacologists; (ii) PlantPAD provides download functionality for some ML-based models, but lacks specific introductions to these models, and if detailed information on these models could be added, it would attract more users from the bioinformatics field and (iii) PlantPAD currently lacks a comprehensive analysis of data, such as statistical charts, tables and so on. If diverse data statistics and analysis could be added, users could more intuitively and quickly understand the distribution and development trends of plant diseases. Thus, to further enhance the value of PlantPAD and attract more users in different fields, it is necessary to continuously improve and the update data and provide more detailed information so that users can better understand and use PlantPAD.

Although the PlantPAD website has significant features, there some areas remain for improvement. For instance, (i) currently, all images in PlantPAD are currently static images. However, if virtual reality technology is utilized, we could provide more intuitive and vivid displays of plant diseases in the future. (ii) The current PlantPAD does not support user interaction and socialization, including user comments, sharing, discussion, or other functions. In future upgrades, PlantPAD could add features for collecting user feedback and suggestions to promote interaction and knowledge sharing among users, which could enable a better understanding of user needs and improvement directions to continuously improve the user experience and the value of the website. (iii) To ensure the accuracy and timeliness of the data on the website, PlantPAD must also continuously update and maintain its data to meet user needs in the field of plant protection. In conclusion, PlantPAD requires continuous improvement and perfection of the website to better serve a wide range of users.

At present, the applications on PlantPAD focus on disease search, diagnosis and prevention, the platform does not support the use of cameras on mobile phones for outdoor photography recognition of diseases. Therefore, there is a need to develop more extensive areas of application. These would include the following: (i) intelligent diagnosis of plant diseases: through ML technology and other technologies, PlantPAD could provide intelligent diagnosis of plant diseases and more accurate protection and treatment plans for agricultural production; (ii) monitoring and early warning of plant diseases: PlantPAD could cooperate with remote sensing technology and Internet of Things technology to reach real-time monitoring and early warning of plant diseases and to improve the efficiency of disease prevention and control; (iii) popular science education on the subject of plant diseases: The PlantPAD library could provide popular science education on plant diseases for the public to improve the general understanding and awareness of plant disease prevention. In sum, PlantPAD has a wide range of fields of applications and great potential; it can provide more support and assistance for the development of agricultural production and environmental protection through continuous improvement and optimization.

## Conclusion and outlook

Here, we describe the construction of PlantPAD and its design for image phenomics analysis of disease in plant science. Our platform contains 421 314 images, 63 crops and 310 diseases. PlantPAD presents more image data, more detailed annotations and richer text information than existing databases in this field, and it serves various multiple disciplines of applications and explorations in plant disease science. We provide three typical cases to illustrate easy-to-use and convenient functions in the diagnosis of plant disease, knowledge teaching, and control strategies. The overarching aim of this prodigious database is to empower its users, facilitating their access to plant disease information in an expeditious and convenient manner while fostering rapid identification, accurate diagnosis and efficacious prevention of plant afflictions. PlantPAD is a unique plant disease database, that supports various disciplines. It provides rich data for computer science fields, including computer vision, natural language processing, and machine learning, enabling intelligent detection and analysis of plant diseases. PlantPAD also provides detailed information for biological fields such as plant pathology, microbiology and molecular biology, advancing the understanding and treatment of plant diseases. Moreover, our platform benefits agronomy by allowing real-time monitoring and management of agricultural production, economy and quality, reducing crop losses and costs.

To better serve research, teaching and practice in the plant field, we will continue to develop and improve our database in the following aspects of optimization and innovation: (i) image phenomics data expansion: we will continue to collect and label plant disease images to build a larger, more comprehensive, and more standard database of plant disease and pest images, covering different crop types, pest types, geographical regions, growing environments and other dimensions; (ii) image phenomics data improvement: we will not only provide a basic description of plant diseases, causes of occurrence and methods of prevention and control, as well as other conventional information, but we will also cover more in-depth knowledge fields, such as flora, physiology and biochemistry, genetic inheritance, resource plants, endangered species, invasive species and so on. We won’t stop until we build a richer, more diverse and more interactive database of plant diseases.

## Supplementary Material

gkad917_supplemental_file

## Data Availability

PlantPAD is freely available at http://plantpad.samlab.cn.
