# Supplementary material for: PlantPAD: a platform for large-scale image phenomics analysis of disease in plant science
Source: Nucleic Acids Res. 2023 Oct 28;52(D1):D1556–68. doi: 10.1093/nar/gkad917 (PMC10767946; doi:10.1093/nar/gkad917)
Supplement: gkad917_supplemental_file [file gkad917_supplemental_file.pdf]

Supplementary Data

Table S1: Detailed information about the plant species in the PlantPAD database. There are 132,837 images of 36 PlantPAD health species, and the table displays the detailed number of images for each plant health species.

Plant Name	Image Number	Plant Name	Image Number	Plant Name	Image Number
blueberry healthy	6390	gauva healthy	1554	hops healthy	528
corn healthy	13460	okra healthy	1877	pongamia pinnata healthy	644
jamun healthy	1558	rice healthy	2488	soyabean healthy	12772
lemon healthy	1318	chinar healthy	1206	sugarcane healthy	488
basil_healthy	1148	grape healthy	6635	bean healthy	428
cherry healthy	8229	cauliflower healthy	1206	jatropha healthy	266
cotton healthy	2016	raspberry healthy	4087	mango healthy	340
pepper healthy	10524	tea healthy	1074	peach healthy	5624
pomegranate healthy	1574	alstonia scholaris healthy	358	tomato healthy	9713
wheat healthy	4718	apple healthy	15274	banana healthy	128
arjun healthy	1440	citrus healthy	87	potato healthy	5168
cucumber healthy	1341	coffee healthy	435	strawberry healthy	6741

Table S2: Detailed information about plant species can be found in the PlantPAD database.There are 288,477 images for the 310 PlantPAD disease categories, and the table displays the detailed number of images for each plant disease category. Due to the high number of data entries in three tables, the table below shows the remaining disease categories.

Plant Name	Disease Name	Number	Plant Name	Disease Name	Number
apple	black rot	4424	mulberry	bud leaf white spot disease	36
apple	round spot disease	25	mung bean	magnesium deficiency	10
apple	powdery mildew	1281	mung bean	brown leaf spot	575
apple	zinc deficiency	16	mung bean	sooty blotch	16
apple	nitrogen deficiency	18	nectarine	bacterial perforation disease	42
apple	cedar apple rust fungus	5313	okra	yellow vein mosaic disease	1072
apple	grey blight	3356	onion	blight	63
apple	brown leaf spot	14	onion	virus disease	20
apple	spotted leaf litter	27	onion	gray mold	25
apple	phosphorus deficiency disease	13	onion	black spot disease	15
apple	phytotoxicity	11	onion	purple spot disease	51
apple	rust	2798	onion	sclerotium diseases	19
apple	scab	13419	onion	downy mildew	50
arjun	leaf spot disease	3450	peach	latent mosaic disease	12
bael	huanglongbing	118	peach	bacterial spot	11485
banana	cordana leaf spot	162	peach	iron deficiency	24
banana	black sigatoka	473	peanut	powdery mildew	53
banana	leaf spot disease	473	peanut	scab disease	11
banana	panama disease	173	peanut	anthracnose	29
bean	powdery mildew	49	peanut	black spot disease	39
bean	rust	436	peanut	scorch disease	43
bean	bacterial leaf spot	432	peanut	brown leaf spot	62
bean	bacterial blight	73	peanut	phosphorus deficiency disease	14
blueberry	leaf spot disease	50	peanut	rust	10
blueberry	iron deficiency	15	peanut	net blotch and brown spot mixture	18
blueberry	red ringspot virus disease	22	peanut	iron deficiency	12
cabbage	phytotoxicity	12	peanut	black spot and scorch spot mixture	10
cauliflower	black rot	100	peanut	black spot and net blotch mixture	10
cauliflower	downy mildew	177	peanut	magnesium deficiency	13
cauliflower	bacterial spot rot	173	peanut	ozone injury	25
celery	leaf spot disease	30	pear tree	sooty blotch	52
celery	bacterial leaf spot	28	pear tree	brown leaf spot	35
celery	phyllosticta leaf spot	50	pear tree	grey blight	10
celery	black spot disease	13	pear tree	rust	61
celery	bacterial leaf blight	23	pepper	umbilical rot	42
celery	phytotoxicity	15	pepper	virus disease	60
celery	spot blight	52	pepper	white spot disease	14
cereal millet	leaf blight	10	pepper	powdery mildew	10
cereal millet	bacterial stripe	10	pepper	blight	64
cereal millet	millet black sheath disease	10	pepper	bacterial spot	7912
cereal millet	powdery mildew	22	pepper	leaf blight	50
cherry	brown spot perforation disease	41	pepper	brown leaf spot	37
cherry	bacterial perforation disease	17	pepper	mildew	23
cherry	mosaic virus	14	pepper	black spot disease	18
cherry	powdery mildew	7364	pomegranate	anthracnose	544
chinar	leaf spot disease	240	potato	late blight	6991
chinese cabbage	black rot	49	potato	early blight	9091
chinese cabbage	grey blight	12	pumpkin	downy mildew	5115
chinese cabbage	blight	19	pumpkin	powdery mildew	5980
chinese cabbage	downy mildew	10	pumpkin	virus disease	38
chinese cabbage	white spot disease	65	radish	white spot disease	23

Table S3: Detailed information about plant species can be found in the PlantPAD database.There are 288,477 images for the 310 PlantPAD disease categories, and the table displays the detailed number of images for each plant disease category. Due to the high number of data entries in three tables, the table below shows the remaining disease categories.

Plant Name	Disease Name	Number	Plant Name	Disease Name	Number
chinese cabbage	bacterial brown spot disease	18	radish	yellow spot leaf blight	27
chinese cabbage	magnesium deficiency	20	radish	wrinkle virus disease	1034
chinese cabbage	anthracnose	14	radish	black rot	36
chinese cabbage	bacterial black spot disease	33	radish	black spot disease	1122
chinese cabbage	virus disease	25	radish	mosaic virus	734
chinese cabbage	black spot disease	26	radish	fertilizer damage	10
chinese cabbage	phytotoxicity	10	rice	plague	1000
chinese toon	brown leaf spot	10	rice	brown leaf spot	613
chinese toon	powdery mildew and rust mixture	25	rice	plague	977
chinese toon	bacterial brown spot disease	49	sesame	leaf spot disease	24
chinese toon	rust	10	sesame	yellow dwarf virus disease	32
citrus	huanglongbing	19314	sesame	leaf blight	17
citrus	black spot disease	311	sorghum	target spot disease	22
citrus	canker	368	sorghum	mosaic disease	13
coffee	sooty blotch	257	sorghum	sheath blight disease	25
coffee	rust	683	sorghum	northern leaf blight	18
corn	top rpt	41	sorghum	bacterial stripe	41
corn	zinc deficiency	54	sorghum	anthracnose	16
corn	sheath blight disease	59	sorghum	bacterial red stripe disease	14
corn	northern leaf blight	8909	sorghum	dwarf mosaic virus disease	27
corn	bacterial stripe	60	sorghum	glume mold disease	15
corn	virus disease	29	sorghum	mycosphaerella leaf spot	24
corn	sooty blotch	15	sorghum	gloeocercospora leaf	24
corn	brown leaf spot	48	soybean	phytotoxicity	13
corn	bipolaris maydis	34	soybean	nitrogen deficiency	33
corn	bacterial red stripe disease	60	soybean	grey blight	39
corn	dwarf mosaic virus disease	49	soybean	blight	27
corn	phosphorus deficiency disease	31	soybean	rust	49
corn	blight	1338	soybean	alternaria black spot disease	20
corn	curvularia leaf spot	53	soybean	bacterial spot	62
corn	rust	11077	soybean	mosaic disease	28
corn	red leaf	59	soybean	phytotoxicity	45
corn	genetic stripes	30	soybean	anthracnose	39
corn	kaliopenia	39	soybean	target spot disease	70
corn	root rot	20	soybean	virus disease	20
corn	grey blight	5604	soybean	gray mold	11
corn	round spot disease	28	soybean	magnesium deficiency	50
corn	bacterial wilt disease	63	soybean	downy mildew	533
corn	sunscald	16	soybean	sooty blotch	16
cotton	anthracnose	34	soybean	leaf blight	27
cotton	leaf burning disease	15	soybean	sunscald	11
cotton	blight	29	spinach	black spot disease	29
cotton	eyespot	10	strawberry	calcium deficiency	805
cotton	phytotoxicity	45	strawberry	white leaf spot disease	37
cotton	stem blight	14	strawberry	scorch disease	7763
cotton	bacterial leaf spot	38	strawberry	leaf blight	38
cotton	magnesium deficiency	15	strawberry	brown leaf spot	35
cotton	verticillium wilt	38	sugarcane	red rot	491
cotton	kaliopenia	28	sunflower	bacterial leaf spot	46
cowpea	spot blight	50	sunflower	gray mold	72

Table S4: Detailed information about plant species can be found in the PlantPAD database.There are 288,477 images for the 310 PlantPAD disease categories, and the table displays the detailed number of images for each plant disease category. Due to the high volume, data was recorded in three tables.

Plant Name	Disease Name	Number	Plant Name	Disease Name	Number
cowpea	rust	17	sunflower	leaf scars	140
cowpea	mosaic virus	22	sunflower	black spot disease	28
cowpea	red spot disease	13	sunflower	downy mildew	120
cowpea	gray mold	20	sweet potato	bacterial wilt disease	29
cowpea	shrink virus disease	22	sweet potato	leaf spot disease	58
cowpea	sooty blotch	13	sweet potato	virus disease	23
cucumber	black spot disease	57	sweet potato	magnesium deficiency	502
cucumber	wrinkle virus disease	10	sweet potato	phytotoxicity	11
cucumber	anthracnose	65	sweet potato	frost damage	18
cucumber	downy mildew	60	sweet potato	sooty blotch	1031
cucumber	powdery mildew	51	sweet potato	cercospora leaf spot	17
cucumber	target spot disease	63	sweet potato	scab	20
cucumber	bacterial leaf spot	360	tea	grey blight	100
cucumber	angular leaf spot disease	48	tea	anthracnose	100
cucumber	white spot disease	44	tea	bird eye spot	100
ginger	white spot disease	16	tea	algal leaf spot	113
ginger	anthracnose	31	tea	white spot disease	142
ginger	leaf roll	15	tea	brown leaf spot	113
ginger	mosaic virus	11	tea	red leaf spot	143
ginger	bacterial leaf spot	23	tobacco	virus disease	38
ginger	phytotoxicity	15	tobacco	mosaic disease	47
grape	leaf blight	7251	tobacco	scorch disease	21
grape	phytotoxicity	22	tobacco	ragged leaf spot disease	33
grape	black rot	8386	tobacco	mosaic virus	29
grape	black measles	8950	tobacco	frog eye disease	35
grape	anthracnose	48	tobacco	potato y virus disease	27
grape	leaf roll virus disease	20	tobacco	leaf spot disease	25
grape	anthracnose	60	tobacco	brown leaf spot	46
grape	fan leaf virus disease	19	tomato	leaf spot disease	61
guava	leaf blight	284	tomato	early blight	7975
hawthorn	rust	28	tomato	bacterial spot	10855
hawthorn	leaf spot disease	42	tomato	late blight	10576
hops	powdery mildew	106	tomato	gray leaf spot disease	38
hops	downy mildew	166	tomato	target spot disease	8780
jamun	leaf spot disease	690	tomato	spot blight	42
jujube	rust	11	tomato	septoria leaf spot	8324
kiwifruit	brown leaf spot	13	tomato	yellow leaf curl virus	21120
kiwifruit	mosaic virus	11	tomato	leaf mold	7742
kiwifruit	grey blight	11	tomato	mosaic virus	5704
kiwifruit	leaf spot disease	25	walnut	black spot disease	23
leek	gray mold	622	wheat	mosaic disease	41
leek	fertilizer damage	11	wheat	crustose leaf blight	31
leek	hail damage	714	wheat	brown rust	912
lemon	canker	154	wheat	stripe leaf blight	24
lentil	leaf spot disease	26	wheat	yellow stripe rust	12708
lentil	white spot disease	13	wheat	stripe mosaic disease	48
lentil	brown leaf spot	20	wheat	spindle streak mosaic disease	19
lentil	virus disease	14	wheat	septoria blotch	97
mango	anthracnose	424	wheat	spot blight	47
melon	downy mildew	60	wheat	powdery mildew	27
melon	powdery mildew	66	wheat	bacterial leaf blight	10
mulberry	sooty blotch	18	wheat	leaf blight	43
mulberry	brown leaf spot	40	wheat	yellow spot leaf blight	50
mulberry	anthracnose	17	zucchini	powdery mildew	43
